# Differential Rates of Glycation Following Exposure to Unique Monosaccharides

**DOI:** 10.3390/ijms25136921

**Published:** 2024-06-25

**Authors:** Derek M Clarke, Andrew P Koutnik, Richard J Johnson, Janine M DeBlasi, Benjamin T Bikman, Juan A Arroyo, Paul R Reynolds

**Affiliations:** 1 Department of Cell Biology and Physiology, Brigham Young University, Provo, UT 84602, USA; 2 Sansum Diabetes Research Institute, Santa Barbara, CA 93105, USA; 3 Department of Medicine, University of Colorado, Aurora, CO 80309, USA; 4 Department of Molecular Pharmacology and Physiology, Morsani College of Medicine, University of South Florida, Tampa, FL 33620, USA

**Keywords:** glucose, fructose, allulose, glycation

## Abstract

A complication of reducing sugars is that they can undergo Maillard chemical reactions, forming advanced glycation end-products (AGEs) that can induce oxidative stress and inflammation via engagements with the main receptor for AGEs (RAGE) in various tissues. Certain sugars, such as glucose and fructose, are well known to cause AGE formation. Recently, allulose has emerged as a rare natural sugar that is an epimer of fructose and which is of low caloric content that is minimally metabolized, leading to it being introduced as a low-calorie sugar alternative. However, the relative ability of allulose to generate AGEs compared to glucose and fructose is not known. Here we assess the accumulation of AGEs in cell-free, in vitro, and in vivo conditions in response to allulose and compare it to glycation mediated by glucose or fructose. AGEs were quantified in cell-free samples, cell culture media and lysates, and rat serum with glycation-specific ELISAs. In cell-free conditions, we observed concentration and time-dependent increases in AGEs when bovine serum albumin (BSA) was incubated with glucose or fructose and significantly less glycation when incubated with allulose. AGEs were significantly elevated when pulmonary alveolar type II-like cells were co-incubated with glucose or fructose; however, significantly less AGEs were detected when cells were exposed to allulose. AGE quantification in serum obtained from rats fed a high-fat, low-carb (HFLC) Western diet for 2 weeks revealed significantly less glycation in animals co-administered allulose compared to those exposed to stevia. These results suggest allulose is associated with less AGE formation compared to fructose or glucose, and support its safety as a low-calorie sugar alternative.

## 1. Introduction 

Today the intake of added sugars can amount to 15 to 20 percent of total caloric intake which is associated with increased risk for metabolic disorders including obesity, diabetes, and fatty liver. Both table sugar (sucrose) and high fructose corn syrup (HFCS) contain fructose and glucose which have been mechanistically linked with the development of metabolic syndrome and the exacerbation of related symptoms. Both can result in the extensive production of advanced glycation end-products (AGEs) that can exert both proinflammatory effects as well as oxidative stress via the activation of their transmembrane pattern recognition receptor, the receptor for advanced glycation end-products (RAGE) [1].

Recently a natural rare sugar known as allulose (also referred to as psicose, d-psicose, d-allulose, or pseudo-fructose) has been introduced as a low-calorie alternative to sucrose and HFCS and exhibits about 70% of the sweetness of sucrose. Allulose is a monosaccharide with a molecular formula of C_6_H_12_O_6_ and an epimer of fructose [2] that is minimally metabolized yet not metabolized by fructose-metabolizing enzymes such as fructokinase (unpublished). Rather, allulose may have salutary effects such as the stimulation of glucagon-like peptide-1 (GLP1) and by lowering postprandial glucose [3]. 

One question that has not been addressed is whether allulose may result in significant AGE formation. The unique metabolic profile of allulose, combined with its physiological functions, supports its growing use as a dietary sugar substitute, offering an alternative without compromising sweetness. Nevertheless, whether allulose can generate AGEs has not been well studied.

AGEs are compounds formed via non-enzymatic glycation reactions involving the carbonyl group of reducing sugars and free amino groups of proteins, lipids, or nucleic acids. These reactions can occur both exogenously through dietary sources and endogenously when they form within organs and tissues [4]. Among the various pathways through which AGEs are synthesized, the Maillard reaction is particularly prominent. This process begins with the reaction of reducing sugars such as glucose and xylose with amino groups from macromolecules, forming reversible Schiff bases [5]. These bases are then transformed via Amadori rearrangement into more stable structures known as early glycation or Amadori products. Progressive chemical modifications of these Amadori products via processes of oxidation, reduction, dehydration, condensation, fragmentation, and cyclization lead to the formation of irreversible and complex AGEs [6]. 

The first observation that AGEs are potentially deleterious was during the initial characterization of elevated hemoglobin A1c, which increases with poor glucose control in diabetics [7]. Hemoglobin A1c is an Amadori product formed as a ketoamine resulting from reactions between glucose and the free amino group on the Val residue of the hemoglobin beta chain [8]. AGEs have since been observed to accumulate in various tissues, particularly under conditions of hyperglycemia and oxidative stress, and are implicated in the pathogenesis of numerous age-related diseases [9]. Specifically, AGE accrual has been linked to the development and progression of diabetes, chronic kidney disease, tumors, memory loss, eye disorders, polycystic ovary syndrome, cardiovascular complications, bone-related ailments, periodontitis, and erectile dysfunction [10]. The pathophysiological mechanisms associated with AGEs encompass two principal aspects: firstly, the crosslinking of AGEs with macromolecular entities such as proteins and nucleic acids, which results in structural and functional alterations, thereby precipitating tissue damage; secondly, causing inflammation via the binding of AGEs to specific cell surface receptors, notably the receptor for advanced glycation end-products (RAGE). AGE–RAGE interactions promote cellular and tissue disruption through indirect modifications of cellular architecture and functionality, primarily via the activation of inflammatory and oxidative stress responses [11,12]. Such pro-inflammatory modalities are predominantly mediated through NF-κB signaling pathways [13]. These combined actions illustrate the complex role of AGEs in disrupting cellular and tissue homeostasis, contributing significantly to the etiology of various chronic diseases. Therefore, the accumulation and formation of AGEs are critical in glycation-related molecular aging and disease progression, underscoring the need for interventions that either reduce AGE concentrations or mitigate their biological effects. Therefore, we investigated whether allulose might induce AGE formation and compared accrual to glycation mediated by fructose or glucose in cell-free and cell culture systems as well as in in vivo models.

## 2. Results

**Figure 1:**
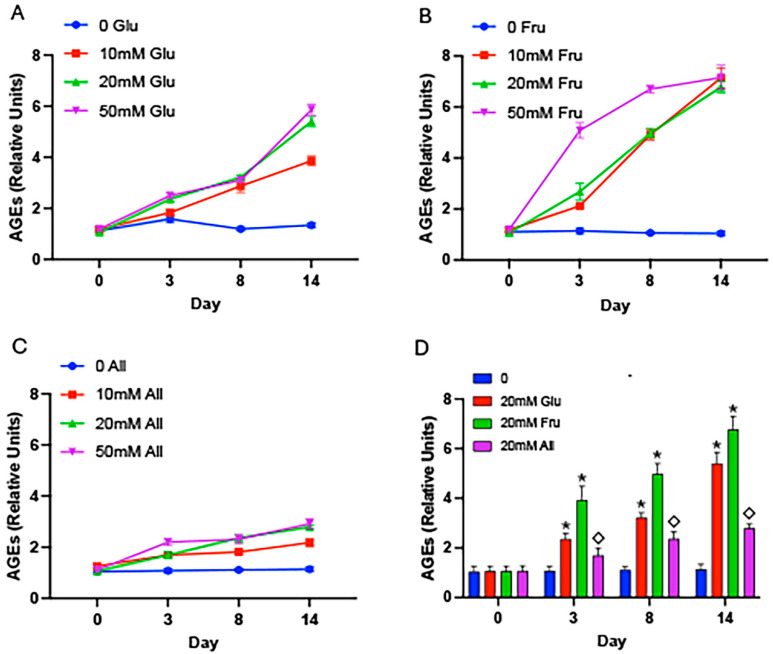
AGEs in cell-free conditions. BSA and glucose (**A**), fructose (**B**), or allulose (**C**) were incubated for 3, 8, or 14 days and AGEs were quantified by ELISA. AGE accrual was elevated after just 3 days with glucose and levels continued to increase through 14 days (**A**). Fructose caused marked increases in AGE accrual at 3, 8, and 14 days compared to controls and 10 mM, 20 mM, or 50 mM fructose led to a common ceiling of AGE formation regardless of concentration (**B**). Allulose caused only modest AGE formation regardless of concentration or timespan (**C**). A comparison of 20 mM glucose, 20 mM fructose, and 20 mM allulose revealed significant increases in glycation in glucose and fructose groups compared to controls and significantly less glycation in allulose samples compared to glucose or fructose samples (**D**). Experiments were conducted in quadruplicate and statistically different values are noted as *p* ≤ 0.05; (*) designates comparisons between 20 mM glucose or 20 mM fructose and controls and (◇) notes comparisons between allulose and glucose or fructose.

As glycation occurs when reducing sugars and proteins interact, we initially sought to assess AGE accumulation when these two key molecules were present in a cell-free system. BSA was incubated with either glucose, fructose, or allulose for up to 14 days. We discovered a significant dose-dependent increase in glycated BSA in the presence of glucose where 50 mM glucose resulted in the highest level of glycation (Figure 1A). Fructose-mediated glycation peaked at 14 days regardless of concentration (Figure 1B). We observed only modest glycation with allulose over the time course (Figure 1C), which when compared to 20 mM glucose or fructose, resulted in significantly less AGE formation (Figure 1D).

**Figure 2:**
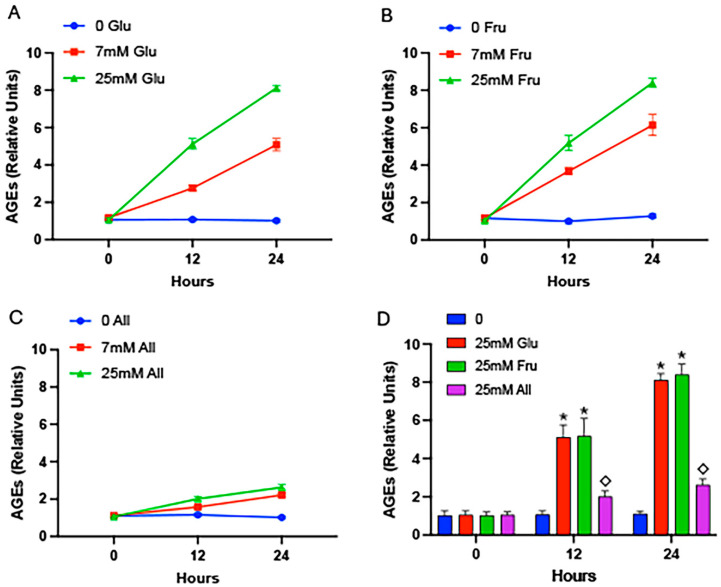
AGEs in conditioned cell culture media. Media was procured from cells exposed to glucose (**A**), fructose (**B**), or allulose (**C**) at 0 h, 12 h, or 24 h and AGEs were quantified by ELISA. Compared to 0 h samples, AGE accrual was elevated at 12 h and 24 h with glucose or fructose regardless of concentration (**A**,**B**). Allulose elicited only modest AGE formation regardless of concentration or duration (**C**). A comparison of 25 mM glucose, 25 mM fructose, and 25 mM allulose revealed significant increases in glycation in media from cells exposed to glucose and fructose compared to controls and significantly less glycation in allulose media samples compared to glucose or fructose (**D**). Experiments were conducted in quadruplicate and statistically different values are noted as *p* ≤ 0.05; (*) designates comparisons between 25 mM glucose or 25 mM fructose and controls and (◇) notes comparisons between allulose and glucose or fructose.

**Figure 3:**
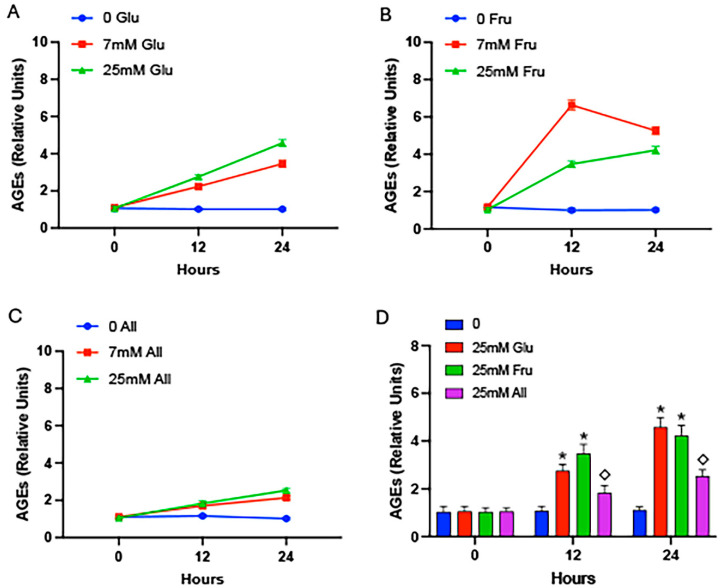
AGEs in cell lysates. Cell lysates were procured following exposure to glucose (**A**), fructose (**B**), or allulose (**C**) at 0 h, 12 h, or 24 h and AGEs were quantified by ELISA. Compared to 0 h samples, AGE accrual was elevated at 12 h and 24 h with glucose regardless of concentration (**A**). A concentration of 7 mM fructose led to peak AGE formation at 12 h, and a decrease, although still significantly higher than 0 mM controls, at 24 h (**B**). Allulose elicited only modest AGE formation regardless of concentration or duration (**C**). A comparison of 25 mM glucose, 25 mM fructose, and 25 mM allulose revealed significant increases in intracellular glycation in cells exposed to glucose and fructose compared to controls and significantly less glycation in allulose-exposed cells compared to glucose or fructose (**D**). Experiments were conducted in quadruplicate and statistically different values are noted as *p* ≤ 0.05; (*) designates comparisons between 25 mM glucose or 25 mM fructose and controls and (◇) notes comparisons between allulose and glucose or fructose.

Cells exposed to glucose resulted in significantly elevated AGEs in conditioned media, after either 12 h or 24 h in culture (Figure 2A). Glycation was similarly elevated when cells were exposed to fructose (Figure 2B). As observed in the cell-free model (Figure 1C), cells exposed to allulose did not elicit marked AGE formation in conditioned media (Figure 2C). A comparison of media from all experimental groups was next pursued wherein cellular exposure to 25 mM glucose, fructose, or allulose was compared. These analyses revealed significantly more AGE accrual in culture media from cells exposed to glucose or fructose when compared to monosaccharide-free media controls (Figure 2D). Notably, the allulose-exposed cells culminated in significantly less AGE formation in the media compared to media procured from the glucose- or fructose-exposed cells (Figure 2D). An analysis of cell lysates was next performed to quantify intracellular AGE formation. We discovered significantly elevated glycation in cells exposed to glucose (Figure 3A), yet intracellular AGE quantities were less abundant when compared to extracellular AGEs following glucose exposure (Figure 2A). Interestingly, cells exposed to 7 mM fructose experienced a steep increase in intracellular glycation at 12 h that decreased at the 24 h time point (Figure 3B). Allulose exposure only modestly accentuated intracellular glycation (Figure 3C). When all cell lysates were compared, 20 mM glucose or 20 mM fructose led to significantly more AGE accumulation compared to controls and 20 mM allulose led to significantly less intracellular AGE formation compared to glucose or fructose (Figure 3D).

**Figure 4:**
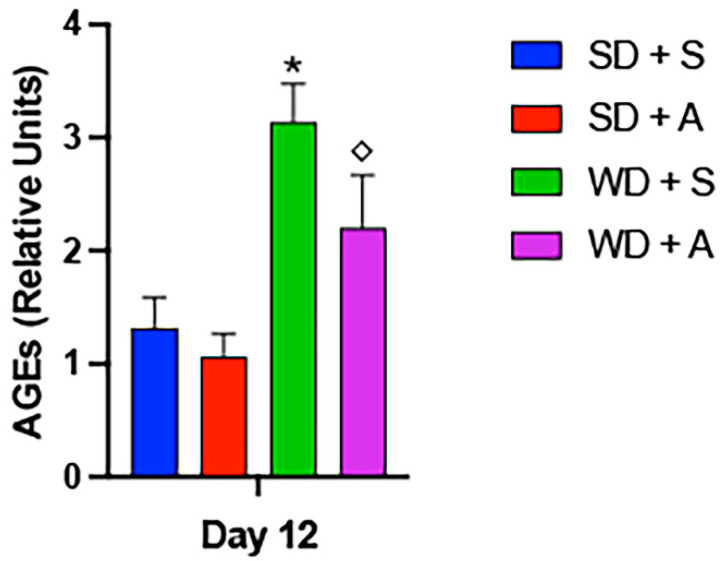
AGEs in rat serum. Total AGEs were detected in rat serum following 14 days of standard diet (SD) + stevia, Western diet (WD) + stevia, SD + allulose, or WD + allulose. There were no glycation differences between rats fed the standard diet. AGE formation was significantly elevated in rat serum following Western diet exposure and notably, Western diet-mediated glycation was significantly decreased with allulose compared to stevia. Experiments involved *n* = 10 rats and statistically different values are noted as *p* ≤ 0.05; (*) designates comparisons between SD + stevia and WD + stevia and (◇) notes comparisons between WD + stevia and WD + allulose.

Because cell-free and in vitro analyses suggested allulose caused less glycation compared to glucose or fructose (Figure 1, Figure 2 and Figure 3), we sought to investigate whether allulose resulted in less systemic AGE accrual in a rodent model. Rats were accordingly fed a standard rodent chow or HFLC Western chow supplemented with either stevia or allulose. After 14 days, rat serum was evaluated for AGE abundance. We found that there was no change in glycation in rats fed standard chow that received stevia or allulose in the drinking water (Figure 4). However, systemic glycation products were significantly increased in animals receiving Western diet compared to standard diet, although allulose significantly decreased Western diet-mediated glycation compared to stevia. (Figure 4). 

## 3. Discussion

Clinical trials in individuals with T1DM (Type 1 diabetes mellitus) and T2DM (Type 2 diabetes mellitus) have demonstrated that hyperglycemia is a significant contributor to the pathogenesis of notable complications including nephropathy, retinopathy, neuropathy, and accelerated atherosclerosis [14]. Research of this variety emphasizes that hyperglycemia is an independent risk factor for these vascular complications [15,16]. While multiple pathways have been identified in efforts to elucidate the mechanisms by which hyperglycemia induces diabetic pathologies [17], an emerging mechanism of consequence is the formation of advanced glycation end products (AGEs) [18].

In addition to chronic hyperglycemia, the administration of reducing sugars such as glucose and fructose may also result in AGE formation. Indeed, all reducing sugars [19], as well as certain sugar-related molecules such as ascorbic acid, can initiate the Maillard reaction in vivo. The slow reaction rate of glucose with proteins led to the belief that AGEs form primarily in long-lived extracellular molecules. Recently, the rapid intracellular formation of AGEs from various macromolecular precursors has garnered added attention [20]. While glucose exhibits the slowest glycation rate among intracellular sugars, the glycation rate is directly proportional to the percentage of sugar in the open-chain form, with fructose reacting 7.5 times faster than glucose (0.002% open-chain form) [21,22]. Strikingly, the glycolytic intermediate glyceraldehyde-3-phosphate (a molecule that exists as a 100% open-chain form) results in over 200 times more glycated protein compared to equimolar amounts of glucose alone [22]. Consequently, intracellular Maillard reactions are significantly influenced by precursors other than glucose, such as fructose and its metabolites—glyceraldehyde, dihydroxyacetone phosphate, and glyceraldehyde-3-phosphate—and the dicarbonyl compounds methylglyoxal and 3-deoxyglucosone. For instance, a classic study investigated the formation of 3-deoxyglucosone in Maillard reactions involving glucose and fructose [23]. It was discovered that 3-deoxyglucosone is a major carbonyl intermediate, with fructose showing higher reactivity than glucose, leading to significant protein polymerization and cross-linking via glycation [23]. A subsequent study focused on the role of highly reactive triose sugars like glyceraldehyde and glyceraldehyde-3-phosphate in protein cross-linking during the perpetuation of Maillard chemical reactions. The study identified new cross-linking compounds formed by these trioses, highlighting their significant impact on protein modification [24]. Finally, research by Suzuki et al. examined the cytotoxic effects of methylglyoxal and 3-deoxyglucosone, two key dicarbonyl intermediates of the Maillard reaction, on cells. Their research demonstrated that these compounds are highly reactive and contribute to the modification and cross-linking of glycated proteins, leading to subsequent cellular dysfunction [25]. These reactive glycolytic intermediates are believed to be key contributors to intracellular AGE formation due to their higher reaction rates. Supporting these observations are data indicating 10 to 20% of the sugar moieties attached to human ocular lens proteins are linked via carbon 2, highlighting the role of endogenous fructose in the glycation process in vivo [26]. Our discoveries that glucose and fructose lead to excessive glycation in cell-free, in vitro, and in vivo conditions add substantially to the narrative that details a propensity for glycation by these prevalent monosaccharides [27]. For example, numerous studies have demonstrated that both glucose and fructose contribute to excessive glycation in various conditions, with fructose often being more potent in inducing glycation-related damage. Research by Semchyshyn et al. compared the glycoxidation effects of glucose and fructose using both in vitro and in vivo models involving Saccharomyces cerevisiae [28]. It was found that fructose led to higher levels of autoxidation and glycation products in vitro compared to glucose. However, they discovered via in vivo experiments that there was no significant difference in glycoxidation product levels between glucose and fructose exposure. The study also highlighted a role for antioxidant and antiglycation enzymes in mitigating glycoxidation stress [28]. A more recent study by Mou et al. compared the glycation abilities of ribose, fructose, and glucose under physiological conditions using BSA. It was observed that ribose and fructose produced more AGEs than glucose. The study specifically demonstrated that fructose glycation led to significant protein conformational changes and was more cytotoxic compared to glucose [29]. The current investigation reinforces these key initial observations and adds additional context via the parallel assessments of allulose-mediated glycation. 

Allulose has more recently demonstrated anti-hyperglycemic, anti-hyperlipidemic, and anti-inflammatory properties. Experimental studies have shown that allulose is effective against obesity and T2DM in both normal and T2DM rat models [30,31], as well as in clinical trials involving healthy [32] and borderline diabetic individuals [33]. Investigations into the mechanisms of its glucose-lowering effects have explored its preferential intestinal absorption over glucose and its inhibition of the enzymatic activities of glucoamylase and maltase. For example, a recent study investigated the absorption mechanism of d-allulose, demonstrating that d-allulose is absorbed via GLUT5 transporters in the small intestine, which is distinct from pathways through which glucose is absorbed. This preferential absorption mechanism highlighted how allulose may modulate postprandial glucose levels in the organism [34]. Related research expanded the role allulose may play in the mitigation of glucose impact via the functions of key enzymes. Such endeavors explored the inhibition profiles of maltase-glucoamylase and sucrase-isomaltase, providing insights into modalities whereby allulose could inhibit these enzymes, thus reducing glucose absorption and postprandial glucose levels [35].

In the current research we investigated whether allulose can induce AGE formation and how the degree of glycation compares with fructose and glucose. We found that allulose was associated with only mild AGE formation compared to fructose and glucose. Furthermore, allulose was associated with less AGE formation in the setting of a Western diet than what was observed with stevia. These observations suggest that allulose may actually be associated with reduced AGE formation in the setting of a Western diet and is consistent with an additional beneficial effect of this natural low-calorie sugar. This study is consistent with previous work published by Hossain et al. that established functions for allulose in obesity and glycemic control in T2DM OLETF (Otsuka Long-Evans Tokushima Fatty) rats [36]. A subsequent study further demonstrated that allulose may have an effective role in providing antioxidant contributions via Maillard reaction products [37]. 

While our data demonstrate diminished glycation by allulose, key subsequent studies are essential in order to further clarify the impact of glycation orchestrated by these three monosaccharides. For instance, research by ourselves and others have shown distinct inflammatory and oxidative stress burdens perpetuated by AGEs via interactions with their most common receptor, RAGE [38,39,40]. A follow-up undertaking is critical wherein the AGEs generated by glucose, fructose, and allulose are exhaustively characterized as to the extent of inflammation that results from interactions with RAGE. Although these considerations were not entertained in the current endeavor, assessing the true physiological impact of glucose, fructose, and allulose during glycation should include answers to such hypothesis-driven questions. In particular, critical assessments that remain to be completed involve the direct evaluation of NF-κB-mediated inflammation, imbalances between oxidants and antioxidants, and the de novo function of glycated targets. Although appropriately deemed a limitation to the current research, subsequent research endeavors should assess these mechanistic outcomes in order to clarify cellular dysfunction that results from glycation.

## 4. Materials and Methods

### 4.1. Cell-Free Experiments

Glass tubes were used for cell-free experiments, each filled with 1 mL of 5% bovine serum albumin (BSA) in PBS. Tubes were supplemented, in quadruplicate, with 0 mM (PBS only), 10 mM, 20 mM, or 50 mM glucose (Cat# AAA1682836, Fisher Scientific, Waltham, MA, USA), fructose (Cat# AAA177180B, Fisher Scientific), or allulose (AllSWEET^®^, Anderson Advanced Ingredients, Irvine, CA, USA). After capping with aeratable caps, tubes were incubated at 37 °C and subjected to vigorous shaking at 200 rpm for 3, 8, or 14 days. 

### 4.2. Cell Culture Experiments

Immortalized A549 cells, which are pulmonary alveolar type II-like cells originally derived from human lung adenocarcinoma, were obtained from American Type Culture Collection (ATCC, Manassas, VA, USA, Cat# CCL-185) and used in this study. Cells were maintained and cultured in RPMI medium (Mediatech, Manassas, VA, USA) supplemented with 10% fetal bovine serum (FBS) and 1% penicillin and streptomycin. Following subculture, cells were plated into 6-well plates and provided glucose-free media (0 mM glucose) for 6 h. Control cells in quadruplicate were maintained in 0 mM glucose media for the duration of the experiment. Experimental groups involved cells exposed to 7 mM or 25 mM glucose (Cat# AAA1682836, Fisher Scientific), fructose (Cat# AAA177180B, Fisher Scientific), or allulose (AllSWEET^®^, Anderson Advanced Ingredients) and comparisons were made between these experimental groups and control cells exposed to carbohydrate-free media. Media and cell lysates were procured immediately after the initial glucose-free media incubation period (designated as 0 h), and then again in separate cultures after 12 h or 24 h.

### 4.3. Animal Experiments

Twelve-week-old female and male Wistar rats were maintained in a pathogen-free environment under a 12 h light/dark cycle with unrestricted access to food and water. Animals were randomly divided into four experimental groups (*n* = 10; 5 female, 5 male) for a 12-week trial: standard lab chow with stevia, Western diet chow with stevia, standard lab chow with allulose, or Western diet chow with allulose. Western diet was obtained from Research Diets (D12266B, New Brunswick, NJ, USA) and it contained sucrose (290.0 g per 1000 g sample, pr 29.0%), saturated fat (butter, 177.0 g per 1000 g samples or 17.7%), and polyunsaturated fats (corn oil, 215.0 g per 1000 g sample or 21.5%). These ingredients are key components of the classic Western diet which is rich in carbohydrates that deliver glucose immediately via sucrose or in a steady fashion. The fats are also notable for providing energy storage and the absorption of fat-soluble vitamins and essential fatty acids. Stevia was used as a natural non-caloric sweetener control. Stevia and allulose were provided in drinking water, and each animal had access to 30 mL of sterile water daily with either two drops of stevia (sweetened to taste) or 3% allulose (to reach a daily dose of roughly 1.9 g/kg/day), as has been done previously [41]. At the end of the 12 weeks, tail blood was collected and rats were euthanized. Whole blood was inverted 4–5 times and allowed to coagulate for at least 30 min then centrifuged at 1200 RCF for 10 min. The serum supernatant was removed and total AGEs were quantified by ELISA as outlined below. Animal research was approved by Brigham Young University’s Institutional Animal Care and Use Committee (IACUC), conforming to all relevant guidelines, under protocol number 23–1222.

### 4.4. Quantification of AGEs

An AGE-specific ELISA kit was used as directed in the included manufacturer’s instructions (cat # CEB353Ge, Cloud-Clone Group, Katy, TX, USA) to quantify AGEs in cell-free samples, aliquots of conditioned cell culture media, and cell lysates at the various time points outlined above. Blood serum was procured on the date of sacrifice from animals and AGE abundance was quantified with the same molecule-specific ELISA kit (Cloud-Clone Group).

### 4.5. Statistical Analyses

Mean values ± standard deviation were assessed by one or two-way ANOVA, followed with Student’s *t*-tests. Results are representative and those with *p* values < 0.05 were considered significant. Statistical analyses were performed using GraphPad Prism 7.0 software.

## Data Availability

All data gathered in the current investigation are presented within the article. Data and other materials are available from the corresponding author upon reasonable request.
